# Oral Mucositis in Pediatric Patients in Treatment for Acute Lymphoblastic Leukemia

**DOI:** 10.3390/ijerph14121468

**Published:** 2017-11-28

**Authors:** Isabella Lima Arrais Ribeiro, Rebecca Rhuanny Tolentino Limeira, Ricardo Dias de Castro, Paulo Rogério Ferreti Bonan, Ana Maria Gondim Valença

**Affiliations:** 1 Post-Doctorate Researcher in Post-Graduate Program in Dentistry, Universidade Federal da Paraíba, João Pessoa, Paraíba 5045, Brazil; 2 Master student in Post-Graduate Program in Dentistry, Universidade Federal da Paraíba, João Pessoa, Paraíba 5045, Brazil; rebecca.rhuanny@yahoo.com.br; 3 Department of Clinical and Social Dentistry, Universidade Federal da Paraíba, João Pessoa, Paraíba 5045, Brazil; ricardodiasdecastro@yahoo.com.br (R.D.d.C.); pbonan@yahoo.com (P.R.F.B.); anamvalenca@gmail.com (A.M.G.V.)

**Keywords:** lymphoid leukemia, child, adolescent

## Abstract

Oral mucositis in oncologic patients is the most undesirable event of the chemotherapeutic treatment. This study aimed to identify damage to the oral cavity resulting from chemotherapy in pediatric patients with acute lymphoblastic leukemia (ALL). This is a prospective study with a sample of 42 children and adolescents evaluated for 10 consecutive weeks after diagnosis. The modified Oral Assessment Guide (OAG) was used, and data were analyzed by Spearman’s rank correlation coefficient (α = 5%). Changes to the normal lips and saliva were positively related to an increase in the OAG score during all 10 weeks of evaluation. Alterations to the labial mucosa were correlated with an increase in the OAG score from the 2nd to 10th week, which was also found for changes in the tongue and in the swallowing function in Weeks 1, 6, 8, 9, and 10 and for gum changes from the 5th to 7th week. No significant vocal changes were correlated with the total OAG score at any point during the monitoring period. Changes in lips, cheek and/or palatal mucosa, labial mucosa, and gum areas and in swallowing function were positively correlated with an increase in the severity of oral mucositis in patients with ALL after beginning chemotherapeutic treatment.

## 1. Introduction

Leukemia is considered the most prevalent underlying disease in pediatric patients and is characterized by the uncontrollable production of immature leukocytes [1]. Among existing types of leukemia, acute lymphoblastic leukemia (ALL) primarily affects leukocytes and is the most serious and most common type of childhood cancer, accounting for approximately 80% of cases of leukemia from 0 to 19 years of age [2].

Chemotherapy is one of the treatment modalities with the highest probability of curing many tumors, including the most advanced cases, and is the treatment that most increases the survival of cancer patients [1,3,4]. It is also used to rapidly destroy malignant proliferative cells. However, it has the undesirable effect of affecting normal tissues with high mitotic rates, including the oral mucosa, the gastrointestinal tract, and hematopoietic tissue [5,6].

Approximately 40% of cancer patients subjected to chemotherapeutic treatment exhibit oral complications resulting from stomatotoxicity [7] with the common onset of inflammation and ulceration of the oral mucosa (mucositis), which clinically manifests in an edematous, erythematous and friable form, resulting in pain, discomfort, dysphagia, and systemic weakness [8,9], being the most important oral complication resulting from antineoplastic chemotherapy [9].

The present study aimed to identify damage to the oral cavity and functions that occurs during the induction phase of chemotherapy remission for the treatment of acute lymphoblastic leukemia in pediatric cancer patients. 

## 2. Patients and Methods

### 2.1. Study Design, Site, and Population

This is a quantitative and observational study that adopted an inductive approach, a comparative-statistical procedure and an indirect documentation technique. The study was conducted at the Napoleão Laureano Hospital in João Pessoa, Paraíba state (PB), Brazil, from April 2013 to July 2015, and evaluated all patients between 0 and 19 years of age who were diagnosed with acute lymphoid leukemia.

### 2.2. Ethical Considerations

The present study was approved by the Human Research Ethics Committee of the Health Sciences Centre, Federal University of Paraíba, under opinion number 255,900 on 23 April 2013 and all the patients included consented in to participate of the present study.

### 2.3. Sample

Forty-two patients aged 0–18 years old were selected according to the inclusion/exclusion criteria outlined in Table 1 and were evaluated after the diagnosis.

### 2.4. Data Collection

Patients were evaluated weekly over a 10-week period (two and a half months), and an initial evaluation was performed according to the inclusion criteria.

Data were collected in the dental office of the department of pediatrics of the Napoleão Laureano Hospital and in inpatient beds. Evaluations were performed using artificial lighting by one previously calibrated examiner (kappa = 0.87).

The modified OAG was used for data collection (Table 2). This is considered a key oral evaluation guide (functions and structures) recognized by the scientific community for the purpose of evaluating changes in the oral mucosa resulting from antineoplastic treatment using chemotherapeutics [10,11]. This instrument enables the evaluation of 8 items according to scales of oral health impairment, scoring each item from 1 to 3, with 1 representing normal conditions, 2 representing mild-to-moderate changes in epithelial integrity or function, and 3 representing severe impairment.

### 2.5. Data Analysis

Data were analyzed by Spearman’s rank-order correlation and inferential statistics using IBM software SPSS (21.0), IBM Corporate, Armonk, NY, USA), with α = 5%.

## 3. Results

Of the 42 patients analyzed, 54.8% (*n* = 23) were female, with a mean age of 7.1 (±4.7) years (median 5, minimum of 2 and maximum of 18 years of age). The ages at which most patients were diagnosed with malignant neoplasm were as follows: 2 (*n* = 7; 16.7%), 4 (*n* = 8; 19.0%), and 5 (*n* = 4; 9.5%) years.

Table 3 and Table 4 outline the measured items according to their OAG scores, which showed alteration/discomfort in the oral cavity during the 10 weeks of evaluation.

The results in Table 3 and Table 4 demonstrate that, in the period evaluated, inability to speak was observed in only one patient in the 4th week due to severe oral mucositis. Severe changes were observed in the ability to swallow, as well as the buccal mucosa or palate, in the 1st and 4th weeks. There were severe alterations in the tongue in the 1st and in last weeks of follow-up. There were also severe alterations in gums in smaller amounts in the 6th and 10th weeks only. More important alterations occurred in the lips, in saliva, and in the labial mucosa, with an occurrence of severe oral mucositis in a large number of patients, in practically all the evaluation weeks.

The total scores for oral mucositis according to the OAG ranged from 8 to 18 and were highest in the 4th and 6th weeks and lowest in the 1st, 5th, and 7th weeks, when scores ranged from 8 to 12 and from 8 to 13, respectively.

Table 5 shows the results from the analysis of correlation between each OAG item during each week of evaluation as well as the total score obtained for the degree of oral mucositis/impairment.

Changes in lips and saliva were positively correlated with the total OAG score during all study weeks (*p* ≤ 0.05), as were changes in labial mucosa between the 2nd and 10th weeks and changes in swallowing function and the tongue in the 1st, 6th, 8th, 9th, and 10th weeks (Table 5).

## 4. Discussion

The oral monitoring of patients after the diagnosis of a cancer is fundamental for the prevention of complications in systemic conditions during the antineoplastic treatment, especially the stomatotoxic effects of the chemotherapy, including the inflammatory oral lesions and the complications in saliva quantity and the reduction in oral functions such as swallowing and speaking, which more significantly affects quality of life of children and adolescents.

For oral monitoring during treatment, some instruments have been proposed [9,10,11,12]. Our research group selected work as of 2013 that was OAG-modified [11] because the OAG permits the evaluation of important oral sites and functions and includes information about the severity of normality alterations, and these aspects of the OAG have stimulated its use by other researchers of dentistry with respect to oncological patients [13,14]. 

One of the main side effects of antineoplastic therapy with chemotherapeutic agents is oral mucositis, which manifests as mucosal inflammation followed by tissue degeneration caused by the stomatotoxicity of chemotherapeutic drugs. Oral mucositis appears approximately five to seven days after the initiation of antineoplastic therapy and persists over the entire treatment period [11]. These mucosal changes may progress to cellular desquamation, resulting in symptomatic ulcers, with significant effects on the oral function and quality of life of the patient while undergoing treatment and on survival, as therapy may be interrupted due to patient debilitation and/or associated systemic infection [12].

As observed in this study, previous studies have confirmed the predominance of ALL among girls with a mean age of 7.1 years, observing a higher prevalence from 2 to 5 years of age [15,16,17]. The most commonly found skin color of patients was brown, a result similar to that of studies previously conducted in Brazil [17,18].

Our results are in accordance to other studies [11,19] about the discomfort related to the onset of oral cavity lesions was most commonly observed in the initial period of oncologic treatment. 

The voice was impaired in the first six weeks of evaluation, and pain and/or difficulty speaking were the most commonly found symptoms [20] and “pain when swallowing” was most commonly observed from the 8th week onward; this condition is correlated with tissue changes promoted by severe oral mucositis and is related to comorbidities affecting the quality of life and survival of patients during antineoplastic treatment, such as malnutrition due to eating difficulties [21]. This is associated with a concern for the patient’s clinical condition because adequate nutrient intake is fundamental at all stages of antineoplastic treatment [22]. These oral functions are even more severe when associated with a reduction in salivary quantity, which was found in patients in all follow-up weeks.

In the lips, the presence of ulcerations was observed throughout the entire study period (two and a half months), with frequencies ranging from 2.4 to 50.0% of patients. Similarly, the labial mucosa was also impaired by ulcerations between the 2nd and 10th weeks. The patients of this study presented severe oral mucositis in these oral cavity areas in practically all follow-up weeks, and these alterations were positively correlated with an improvement in the total value of the OAG. As the salivary reduction over the entire follow-up study period consequently had a greater impact on the treatment of patients. 

Oral mucositis with tongue impairment may lead to extreme sensitivity and irritation, and, in this study, the tongue was affected throughout treatment, particularly from the 8th week onward, which was associated with a concern for the patient’s clinical condition because the tongue plays key roles in speaking and eating, and mucositis may affect those processes [23,24,25].

The occurrence of lesions in oral mucosa is associated with a poor quality of life of patients during the treatment. Mucositis lesions often interrupt chemotherapy. The chemotherapy cycle cannot continue until these lesions have healed, and this means that these lesions reduce the chance that patients will be cured [21,22,23,24,25,26]. 

Finally, all of these alterations evaluated over the 10 follow-up weeks are difficult to prevent, but they can be detected in their mild and moderate phases, and interventions may prevent injury stop them from becoming severe and impacting quality of life. The success in treatment of this patients depends on a well-functioning team of professionals. 

## 5. Conclusions

The chemotherapeutic treatment of acute lymphoid leukemia in pediatric patients triggers the onset of oral mucositis in the lips, tongue, buccal mucosa or palate, labial mucosa, and gum areas, affects the quality and quantity of saliva, and impairs swallowing function. Those changes may cause imbalances in the oral functions of the stomatognathic system, thereby impairing patients’ health and quality of life, and harm the course of cancer treatment.

## Figures and Tables

**Table 1:** Inclusion and exclusion criteria related to the recruitment of subjects for the present research study.

Inclusion Criteria	Exclusion Criteria
Age from 0 to 19 years	Inflammation of the oral mucosa at the time of diagnosis
Primary diagnosis of acute lymphoid leukemia	Restart of treatment for a recurrent tumor
Treatment at the Napoleão Laureano Hospital by the Unified Health System	Impaired health status
Identification before the initiation of chemotherapy	Failure of the guardian or child/adolescent to sign the informed consent form accepting participation in the study
	Interruption, for any reason, of the monitoring section of the present study

**Table 2:** Oral Assessment Guide (OAG) modified for monitoring the oral health of patients undergoing chemotherapy.

Item	Score		
	1	2	3
Voice	Normal	Deeper or raspy	Difficult talking or painful speech
Swallowing	Normal swallowing	Some pain upon swallowing	Unable to swallow
Lips	Smooth and moist	Dry or cracked	Ulcerated bleeding
Tongue	Pink and moist and papillae present	Coated or loss of papillae with a shiny appearance with or without redness	Blistered or cracked
Saliva	Watery	Thick or ropy	Absent
Mucous membrane (buccal mucosa, palate)	Pink and moist	Reddened or coated (increased whiteness) without ulceration	Ulceration with or without bleeding
Mucous membrane (labial mucosa)	Pink and moist	Reddened or coated (increased whiteness) without ulceration	Ulceration with or without bleeding
Gingiva	Pink and stippled and firm	Edematous with or without redness	Spontaneous bleeding or bleeding with pressure

Source: Cheng, Chang and Yuen (2004); modified with permission from Eilers and colleagues.

**Table 3:** Alteration/discomfort of oral function according to the Oral Assessment Guide scores during the 10 weeks of evaluation.

Item	Change	Weeks									
		1st	2nd	3rd	4th	5th	6th	7th	8th	9th	10th
Voice	Mild/Moderate	*n* = 3 (7.1%)	*n* = 3 (7.1%)	-	*n* = 1 (2.4%)	-	*n* = 1 (2.4%)	-	-	-	-
	Severe	-	-	-	*n* = 1 (2.4%)	-	-	-	-	-	-
Swallowing	Mild/Moderate	*n* = 2 (4.8%)	*n* = 1 (2.4%)	*n* = 1 (2.4%)	*n* = 1 (2.4%)	-	*n* = 2 (4.8%)	-	*n* = 3 (7.1%)	*n* = 3 (7.1%)	*n* = 4 (9.5%)
	Severe	*n* = 1 (2.4%)	-		*n* = 1 (2.4%)	-	-	-	-	-	-
Saliva	Mild/Moderate	*n* = 25 (59.5%)	*n* = 27 (64.3%)	*n* = 29 (69.0%)	*n* = 23 (54.8%)	*n* = 26 (61.9%)	*n* = 19 (45.2%)	*n* = 25 (59.5%)	*n* = 25 (59.5%)	*n* = 30 (71.4%)	*n* = 24 (57.1%)
	Severe	*n* = 3 (7.1%)	*n* = 6 (14.3%)	*n* = 3 (7.1%)	*n* = 7 (16.7%)	*n* = 1 (2.4%)	*n* = 10 (23.8%)	*n* = 5 (11.9%)	*n* = 7 (16.7%)	*n* = 3 (7.1%)	*n* = 3 (7.1%)

Legend: - = did not show alteration.

**Table 4:** Alteration/discomfort in oral mucosa according to the Oral Assessment Guide scores during the 10 weeks of evaluation.

Item	s	Weeks									
		1st	2nd	3rd	4th	5th	6th	7th	8th	9th	10th
Lips	Mild/Moderate	*n* = 13 (31.0%)	*n* = 18 (42.9%)	*n* = 8 (19.0%)	*n* = 8 (19.0%)	*n* = 21 (50.0%)	*n* = 10 (23.8%)	*n* = 8 (19.0%)	*n* = 10 (23.8%)	*n* = 9 (21.4%)	*n* = 11 (26.2%)
	Severe	*n* = 3 (7.1%)	*n* = 7 (16.7%)	*n* = 7 (16.7%)	*n* = 6 (14.3%)	*n* = 4 (9.5%)	*n* = 6 (14.3%)	*n* = 1 (2.4%)	*n* = 9 (21.4%)	*n* = 4 (9.5%)	*n* = 5 (11.9%)
Tongue	Mild/Moderate	*n* = 1 (2.4%)	-	-	*n* = 1 (2.4%)	*n* = 1 (2.4%)	*n* = 2 (4.8%)	-	*n* = 3 (7.1%)	*n* = 4 (9.5%)	*n* = 3 (7.1%)
	Severe	*n* = 2 (4.8%)	*n* = 1 (2.4%)	-	-	-	-	*n* = 1 (2.4%)	-	*n* = 1 (2.4%)	*n* = 1 (2.4%)
Buccal mucosa or palate	Mild/Moderate	-	*n* = 1 (2.4%)	-	*n* = 2 (4.8%)	*n* = 3 (7.1%)	*n* = 1 (2.4%)	*n* = 2 (4.8%)	*n* = 3 (7.1%)	*n* = 3 (7.1%)	*n* = 3 (7.1%)
	Severe	-	*n* = 1 (2.4%)	-	-	*n* = 1 (2.4%)	-	-	-	-	-
Labial mucosa	Mild/Moderate	-	*n* = 2 (4.8%)	*n* = 2 (4.8%)	*n* = 2 (4.8%)	*n* = 2 (4.8%)	*n* = 1 (2.4%)	-	-	*n* = 1 (2.4%)	*n* = 4 (9.5%)
	Severe	-	*n* = 7 (16.7%)	*n* = 3 (7.1%)	*n* = 3 (7.1%)	*n* = 4 (9.5%)	*n* = 6 (14.3%)	*n* = 3 (7.1%)	*n* = 5 (11.9%)	*n* = 5 (11.9%)	*n* = 3 (7.1%)
Gums	Mild/Moderate	*n* = 2 (4.8%)	*n* = 2 (4.8%)	-	-	*n* = 3 (7.1%)	*n* = 5 (11.9%)	*n* = 3 (7.1%)	*n* = 2 (4.8%)	*n* = 2 (4.8%)	*n* = 1 (2.4%)
	Severe	-	-	-	-	-	*n* = 2 (4.8%)	-	-	-	*n* = 1 (2.4%)

Legend: - = did not show alteration.

**Table 5:** Correlations between the total score obtained for oral mucositis and the Oral Assessment Guide items during the 10 weeks of evaluation.

Weeks		Voice	Swallowing	Lips	Tongue	Saliva	Buccal Mucosa or Palate	Labial Mucosa	Gums
1	r.	-	0.424	0.700	0.424	0.853	-	-	0.342
	Sig.	-	0.005	0.000	0.005	0.000	-	-	0.026
2	r.	-	-	0.878	-	0.534	0.380	0.638	-
	Sig.	-	-	0.000	-	0.000	0.013	0.000	-
3	r.	-	-	0.862	-	0.830	-	0.558	-
	Sig.	-	-	0.000	-	0.000	-	0.000	-
4	r.	-	-	0.815	-	0.814	-	0.551	-
	Sig.	-	-	0.000	-	0.000	-	0.000	-
5	r.	-	-	0.759	-	0.508	0.457	0.526	0.463
	Sig.	-	-	0.000	-	0.001	0.002	0.000	0.002
6	r.	-	0.378	0.770	0.378	0.804	-	0.626	0.632
	Sig.	-	0.013	0.000	0.013	0.000	-	0.000	0.000
7	r.	-	-	0.674	-	0.823	0.328	0.467	0.467
	Sig.	-	-	0.000	-	0.000	0.034	0.002	0.002
8	r.	-	0.458	0.904	0.458	0.795	0.458	0.576	0.322
	Sig.	-	0.002	0.000	0.002	0.000	0.002	0.000	0.038
9	r.	-	0.468	0.801	0.548	0.706	0.468	0.536	0.329
	Sig.	-	0.002	0.000	0.000	0.000	0.002	0.000	0.033
10	r.	-	0.531	0.798	0.530	0.789	0.466	0.606	-
	Sig.	-	0.000	0.000	0.000	0.000	0.002	0.000	-

Legend: r. = Spearman’s rank correlation coefficient; Sig. = significance (level of significance=5%); - = did not show a correlation coefficient or the significance was bigger than 5%.
